# Similar, yet different! Comparing Ugandan secondary cities’ food system and nutritional transformations to findings from African primary cities

**DOI:** 10.1186/s42854-022-00047-3

**Published:** 2022-12-28

**Authors:** Heather Mackay, Richard Tusabe, Frank Mugagga

**Affiliations:** 1 Department of Human Geography, Lund University, Lund, Sweden; 2 Geography Department, Umeå University, Umeå, Sweden; 3 Global Initiative for Young Environmental Stewards, Kasangati Town Council, Bulamu Zone A Off Gayaza Road, Wakiso District, P.O. Box 16889, Kampala, Uganda; 4 Department of Geography, GeoInformatics and Climatic Sciences, School of Forestry, Environmental and Geographical Sciences, College of Agricultural and Environmental Sciences, Makerere University, Kampala, Uganda

## Abstract

This research focuses on the food, farming and health experiences of two secondary cities of Uganda (Mbale and Mbarara), comparing findings with studies of primary African cities. We draw from survey data, focus groups with healthcare professionals, and in-depth interviews with varied residents. A feminist geographic perspective explored intersections of food, farming and health with varied aspects of identity, and with place (city itself, but also with rural areas). By comparing our secondary city findings to findings from primary African cities this paper sheds light on whether and how food systems in secondary African cities are transforming, and how urban life at this scale is being experienced. Our analysis suggests a good deal of similarity of food insecurity, dietary diversity, and of non-communicable disease experiences and understandings. The main difference was around the food access strategies, the access to land, and the engagement with agriculture and interaction with the rural. How this might change as these secondary cities grow further is not clear but there should not be an assumption that primary city experiences will inevitably be followed. Our findings offer important insights for future research and for those planning for Ugandan and potentially other African secondary city futures. In comparison to primary SSA cities our findings suggest less advance along theorised nutritional transitions (greater hybridity), a higher relevance of the rural for viable urban lives, yet comparable experience of non-communicable disease. This is intriguing, has implications for theory, and warrants further research.

## Introduction

This research is framed by concerns around the changing food and nutritional environments and a growing multiple burden of malnutrition in cities of the Global South. There has been much discussion of the rapid changes now affecting the ability of urban residents to access food and viable, healthy livelihoods for themselves and their families. How these changes may relate to the growing non-communicable disease (NCD) burden (obesity, diabetes, hypertension) that has been documented across the Global South has been an aspect of debate. The implications of changing food flows in and around cities were further evidenced by the effects of the Covid-19 pandemic, which also revealed serious urban livelihood fragilities in many parts of the world. In this work we were particularly interested to explore the understudied African secondary city scale when much research to date has focused on primary cities. Researchers have noted that it is the secondary cities that are more representative of urban areas within Sub-Saharan Africa (SSA). Moreover, predictions indicate that it will be precisely these secondary cities where the greatest, and the most rapid, growth and change would occur in the coming decades within urban Africa (Parnell and Pieterse 2014; Cohen 2004) [matching the impetus also behind this special issue]. In addition, there have been urgent calls to improve our understanding of the complex links between dietary- and farming-practices, with health and nutrition outcomes in an urban context (Webb 2013; Fan and Pandya-Lorch 2012). Thus, the research (of which this article is a part) sought to investigate the characteristics of two secondary cities of Uganda: Mbale and Mbarara residents’ food, farming and health environments, and to explore how these were being experienced by local residents, using a mix of quantitative, qualitative and spatial methods. In this article we compare the main findings from our body of work against literature from other African cities. Principally we reflect on how the findings from these secondary Ugandan cities were similar to, or different from, previous studies of the capital (Kampala), and other studies of primary cities in Africa. In doing so we address key aspects raised in this special issue call on whether and how food systems in secondary African cities are transforming, how this is experienced, and how findings compare to larger cities. Our findings offer important insights for future research and for those planning for Ugandan and potentially other African secondary city futures.

Next, we outline our methodology before describing the theories that our research engaged with. We then provide a background to our secondary cities–Mbale and Mbarara–before outlining our main findings from these sites to date. After this we engage in the core analytical contribution of this paper where we position these findings in relation to studies of primary African cities, searching to highlight aspects of similarity or difference across primary and secondary SSA cities’ food and nutritional systems. We close by considering possible implications of our comparative analysis.

## Methodology

### The Mbale/Mbarara methods and data

During the project a systematic random sample survey of 1025 Mbale and 970 Mbarara households was conducted where we used internationally validated food security measures such as the household food insecurity access scaled score (HFIASS), household food insecurity access prevalence (HFIAP), and household dietary diversity score (HDDS) as well as socio-economic, incomes, assets and expenditure data. The survey included questions on household farming in rural and urban areas, food transfers from rural- or urban-based relatives or friends, and food sources (traditional markets, small shops, larger supermarkets, street food, own production, remittances/transfers). We also took anthropometric measures of willing adults’ heights and weights to calculate body mass index (BMI) status and we asked about household experience of diagnosed communicable (such as tuberculosis, malaria) and non-communicable (such as diabetes, heart disease, hypertension) diseases. This survey was adapted from the African Food Security Urban Network (AFSUN) with permission. Households were georeferenced–the resulting geographic spread can be seen in the maps in the *Context* section, resulting in good coverage of all residential neighbourhoods. For detailed survey findings see (Mackay et al. 2017).

The second method further explored the dietary and health indications from the household survey with local healthcare professionals using focus group discussions (FGD). These were educated elites responsible for healthcare planning, design or direct patient support across the cities. This work was analysed with a feminist geographic lens and offered important insights into gendered and classed interpretations of body sizes and food-related behaviours. Findings were compared with nutrition transition model ideas, revealing aspects of similarity and difference. Results traced and deconstructed ‘a dominant patriarchal tendency towards blaming women’ (Mackay 2019a, 1) for poor diets and especially for obesity. Yet analysis showed clearly how local discourses are co-created and evolving and thus can be reshaped, which presents, we feel opportunity for positive intervention:

‘findings…clearly show FGD participants constructing identities (the housewife, the provider man), obscuring power relations and failing to recognise the embedded specificity of the patriarchal hetero-normal (Hovorka 2012) of the social context. Yet…debate among the participants, the revelation of assumptions, and the breakdown of these, also reveals the creative co-constructed nature of our depictions of ourselves and of others’ (Mackay 2019a, 16)

The third method of the research involved follow up in-depth individual biographic interviews with some survey respondents, inquiring about their daily life (what they ate, where they went, what they did, challenges they faced). Sampling was purposive but based on cluster analysis within a geographic information system (GIS) of the survey data which identified statistically significant clusters (their occurrence was beyond random chance) of households where people were generally doing better than the average for the town (by the measures of food security, diet diversity, income and health), clusters that were doing less well than average, and clusters that were anomalous for the town. Within these clusters, a box drawn in the GIS selected approximately twenty of these households. From these we then purposively selected about five individuals per cluster for follow-up, aiming for a diversity of gender, age, socio-economic status and farming situation. Final interviews were the result of this process, our ability to re-contact people, and their willingness to participate. This resulted in seventeen interviewees who had been part of the 2015 survey. An additional five were purposively added during fieldwork, three of whom represented a group we classified as ‘salaried elite’ who were underrepresented in the household survey, one met by chance in a food insecure cluster, and one who resided in an anomaly Mbarara cluster that stood out as having particularly different circumstances (turned out to be staff of Mbarara State Prison living in on-site free housing and receiving land-access and seasonal food contributions (at survey time) from their employer). Detailed findings on food sourcing, access strategies and on experiences and interpretations of food, bodies, behaviours were published in (Mackay 2019b) and (Mackay 2022).

### Comparative analysis of Mbale/Mbarara to other studies

The collective findings from these three methods–the household survey; the health professional focus groups; and the biographic interviews with better-off, worse-off, and anomalous individuals–are what we draw upon in this paper to consider our findings in relation to studies from other African cities. We focus on the food security, sourcing and access strategies, involvement in farming, and NCD findings.

Since secondary cities are an emerging, yet still understudied, city scale much of the work published on urban residents’ food, farming and health situation has tended to consider African primary cities. We were particularly interested to see what insights could be gleaned from a comparative reflection across these two urban hierarchies: secondary versus primary. Uganda has only one primary city–that of the capital Kampala (Lwasa 2011). Thus, within the Ugandan context this is the only city available for such comparison. Other studies of urban food environments, double burden malnutrition, the farming activity of urban residents, or studies of NCDs from SSA have often analysed national-level data sets or the larger primary cities, see for example: (Shrimpton and Rokx 2013; Thow 2016; Haggblade et al. 2016; Popkin et al. 2012; Steyn and Mchiza 2014; Thornton 2008). Investigations of urban Africa have generally been from the same cities over again. These tend to be capitals or megacities: Lagos, Dar es Salaam, Addis Ababa, Nairobi, Kampala, Accra, Johannesburg, Cape Town, Lusaka, Harare, Lilongwe; see for example: (Crush et al. 2012; Lee-Smith 2010; Prain 2010; Cole 2008; Egziabher 1994; Prain et al. 2010). The implication in many of these works is that other cities in a country will simply follow the lead of the capital/major cities. It is this body of research that we compare our findings to.

### Rationale surrounding Country and City choices

Much of Uganda’s rapid urban growth in recent years (albeit from a low base) was in its secondary cities, with Mbale growing an average 2% per annum and Mbarara 8.6% per annum (UN-HABITAT 2016). This rate ranks Mbarara within the top five growing cities in Uganda, expanding even faster than Kampala (UN-HABITAT 2016). It is this recent (and projected) growth rate, combined with an acknowledged urbanisation of poverty, and the rising challenges facing cities (service provision, informality, lack of work, pollution) that have focused attention on Ugandan urban issues (UN-HABITAT 2016; Lwasa 2011; Mukwaya et al. 2010).

Uganda is described as a late urbaniser with a speculative urbanism whereby, in the absence of industrial opportunity, people invest in land and real estate as a capital gains strategy (Goodfellow 2017). Demographically, Uganda is young with youth unemployment one of the highest in Africa (Renzaho et al. 2016), presenting both opportunity and serious challenge. The poverty of Ugandan youth is of concern, and the ability to secure work seems more challenging than previously (Datzberger 2018; Reid 2017). These demographic and employment challenges, combined with the urban trends noted above, mean the country houses a potential explosive vulnerability. Research into Ugandan urban livelihoods is thus of real relevance.

Lastly, Uganda is usually described as being in an early stage of food system and nutritional transition (Haggblade et al. 2016) compared to, for example, Kenya or South Africa. Thus, the country may be in the position to learn from other countries’ experiences of urban food security. Policymakers, however, need access to grounded research. There is a need to assess where Uganda’s urban residents are in their food and nutritional environments, and how the psycho-social context shapes attitudes around food, farming and health. Investigating the daily lives of diverse residents, and positioning findings in relation to other SSA urban contexts is necessary and this article makes such a contribution.

Mbale and Mbarara were purposively chosen. There is a size (medium), hierarchical (secondary) and scalar (approximately < 100,000 population) selection here, beyond being understudied. We do not try to claim a holistic view of each city, nor do we assert generalisability to all Ugandan secondary cities. We instead sought to explore these contexts in their specificity and conduct comparative relational analysis (Ward 2008) of varied people’s daily food, farming and health experiences and interpretations. We relate our findings to studies of larger Sub-Saharan African cities.

## Theory frames: urban nutrition and food system transitions and a feminist geographic approach

Our research interrogates conceptualisations of urban food, farming, and epidemiologic transitions described by what is commonly referred to as nutrition transition (Popkin et al. 2012; Popkin 2001). Propositions around how cities develop, their relationship to agriculture, and how peoples’ food purchasing and consumption patterns change with such development are inherent. The dominant idea is that urban lives shift towards a food system and diet pattern focused around large retailer supermarkets, processed and junk foods, eating out, less agriculture (Haggblade et al. 2016; Popkin et al. 2012). These ideas arise out of the modernisation paradigm which theorises how societies evolved from hunter-gather modes of production towards service-based market economies (Nhema and Zinyama 2016; Sanyal 1987), inspired by eighteenth century thinkers such as Adam Smith, and global northern experiences (Peet and Hartwick 2015; Smith 1776 re-edition 1976). Historic northern urban development involved demographic and settlement transitions moving from high fertility-high mortality, more dispersed and rural, largely low-diversity, low-fat, high-complex-carbohydrate, low-meat, low-dairy diets and ways of living towards low fertility-low mortality, more concentrated, urban and sedentary lives characterised by higher-diversity, high-fat diets, with greater meat and dairy consumption (Popkin 1998; Peet and Hartwick 2015; Haggblade et al. 2016). Inherent are assumptions about city morphologies and urban life, and of food and lifestyle change. Often these kinds of urban life evolutions have been statistically and qualitatively associated with indicators of epidemiologic transition from communicable to non-communicable disease (ibid). However, the ‘urban’ is not well-theorised and makes scalar and processual assumptions. It is also important to highlight that many works reporting such nutrition/food-system change draw from country-level aggregated data on crop yields, imports and exports, food balance sheets, gross domestic product (GDP), and national economic data (Popkin 1998, 2001). Such provide a coarse eagle’s eye view. As Nichols notes: ‘research on the nutrition transition has primarily relied on quantitative statistics rendered at national and sub-national levels. While this paints a broad picture of dietary change…these analyses may obfuscate more that they reveal’ (Nichols 2017, 871). She cautions that diet and lifestyle change arise from complex local socio-cultural blend of resources, culture and tradition ‘not readily captured by statistical models’ (Nichols 2017, 871). Nichols argues for more ethnographic study tuned to the ‘nuanced and embodied ways that dietary change’ (Nichols 2017, 873) may actually manifest. Our interviews with diverse urban residents, and FGDs with healthcare professionals take just such ethnographic investigation, complementing the household-level data.

We interpret our findings from a feministic geographic perspective (Hiemstra and Billo 2017; McDowell 1999; Parker 2016). This approach within geography looks to specify, embody and emplace ideas from theory in both the socially constructed, and the material, realities of daily life. For our research, this meant the lives of citizens in the urban spaces of Mbale and Mbarara across various aspects of difference, not only gendered difference. We analysed power geometries and how gendered and classed ideas shaped local interpretations of healthy desirable diets, bodies and food acquisition behaviours. Our further analysis in this paper additionally considers whether our assessment of socio-spatial practices in our secondary Ugandan cities seem to be similar to findings from Kampala, and other African primary cities.

## Context of Mbale and Mbarara, Uganda

The study cities of Mbale and Mbarara are not sites shaped by large slums, or appalling conditions of deprivation, nor are they home to securitised gated communities of wealth, enclaves protected from poverty or criminality. They do not sprawl great distances–you can cross in 5 km. They do not have much vertical growth nor high-rise central districts. They are not sites of gridlocked traffic nor heavy pollution. They are not an apocalypse of extremes (Herrick 2017) like many primary cities. Inequalities do exist, certainly, but perhaps not on the scale of some of the ‘meta-narratives of catastrophe’ (Herrick 2017, 563) evident in some writings on African cities. Mbale and Mbarara are, in our opinion, pleasant towns offering the possibility of an urban environment without some of these more tangible large city challenges, though clear livelihood and food security difficulties do still exist for many.

Mbale Municipality (level that includes surrounding villages, and scale of population census data) was home, with 2016 data, to 92,863 residents and Mbarara Municipality had 195,160 residents (UBoS 2016). However, the actual population of the main city surveyed in our research was lower: estimated 70,000 in Mbale and 90,000 in Mbarara^1^ at data collection. Both sites were not officially cities in Ugandan planning frames but they have now been conferred city status and are designated regional focus cities in line with new urban policy (NPA 2017). Such new status is anticipated to bring Mbale and Mbarara even more rapid growth and investment, likely bringing new pressures but also opportunities. Our work may be of value to those planning for urban Ugandan futures.

Mbale, in the Eastern Region is located towards the Kenyan border by Mount Elgon national park. The town was an important trading centre already prior to colonial times, with intense African, Asian and Arabian merchant activity (Twaddle 1966). These days Mbale is a key administrative centre, experiencing in-migration and rapid growth (Habitat 2011). Housing, employment and servicing problems exist (Silver 2017). The lack of formal job opportunities forces many into casual labour and informal livelihoods (Habitat 2011). Small-scale farming is a common activity of residents, mainly growing staple foods such as maize and cooking bananas [matooke] for consumption (Mugagga et al. (2010)). Coffee is a traditional cash crop (ibid).

**Fig. 1:**
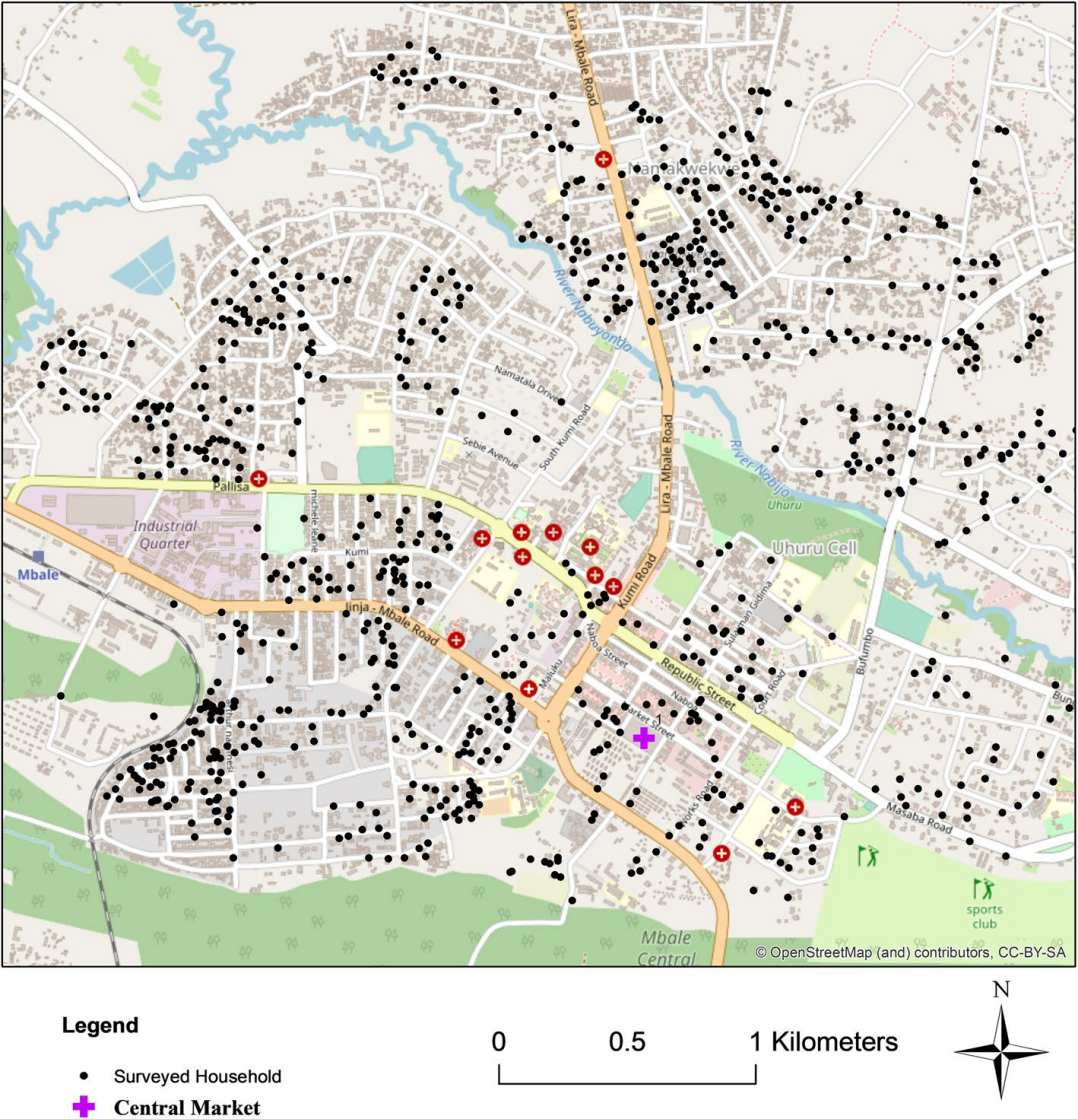
Map of Mbale, Uganda. Dots show the households surveyed. Source: Open street maps. Created by Mackay, H., April 2022

**Fig. 2:**
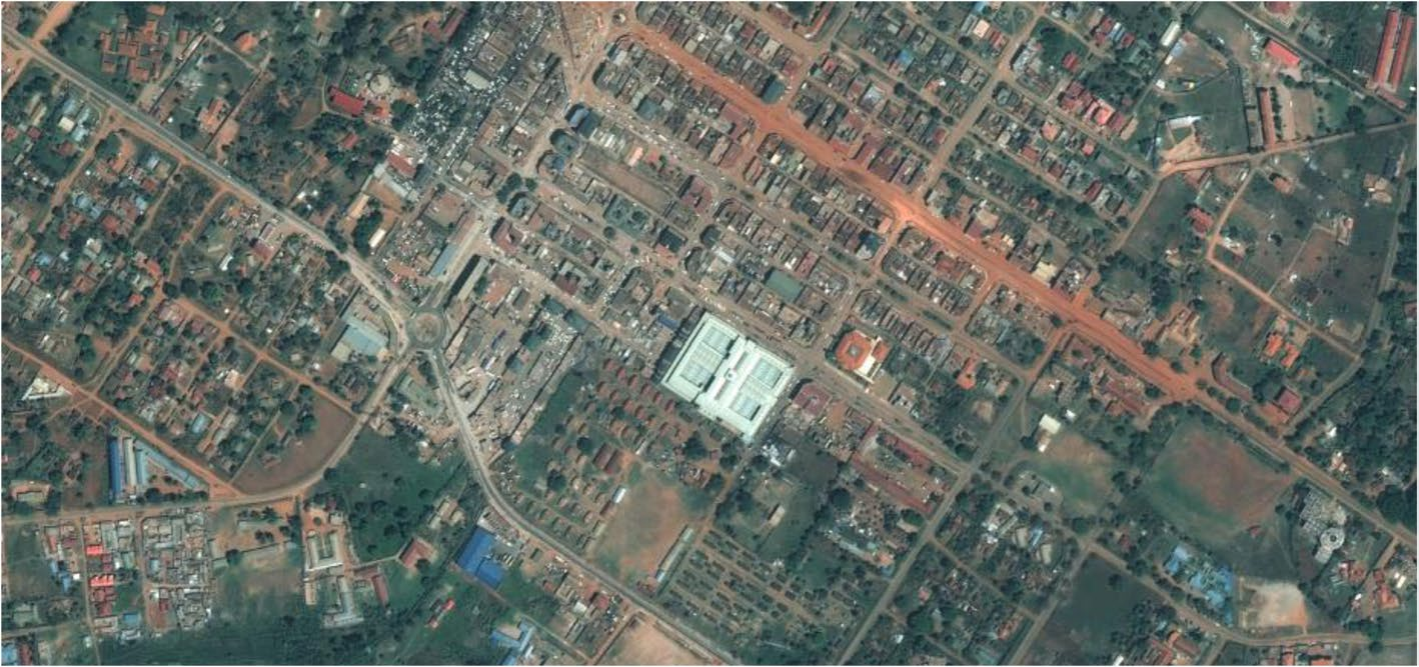
Ordered block layout of Mbale centre. The large structure is the 2016 rebuilt central market hall where the majority of the households purchased their food. A few untarred roads can be seen clearly.Source: Purchased high-resolution satellite imagery © CNES 2014 and 2015, Distribution Astrium Services/Spot Image corporation, USA, all rights reserved. Pleiades Satellite Image of Mbale taken on 8 March 2015

Mbale (together with Jinja) is one of the urban areas to have implemented an original colonial town development plan (Kiggundu (2014). The remains of this town planning, with street grid patterns in the centre, and a colonial elite residential area of large plots (later taken over by Ugandan elite) separated from the disordered African masses by a golf course and green belts (Mukwaya et al. 2010) can still be seen today (Figs. 1 and 2). Colonial plans also instigated ‘altitudinal stratification’ (Mukwaya et al. 2010, 273) where the wealthiest were at the tops of hills, with declining socio-economic status accompanying declining topography.

**Fig. 3:**
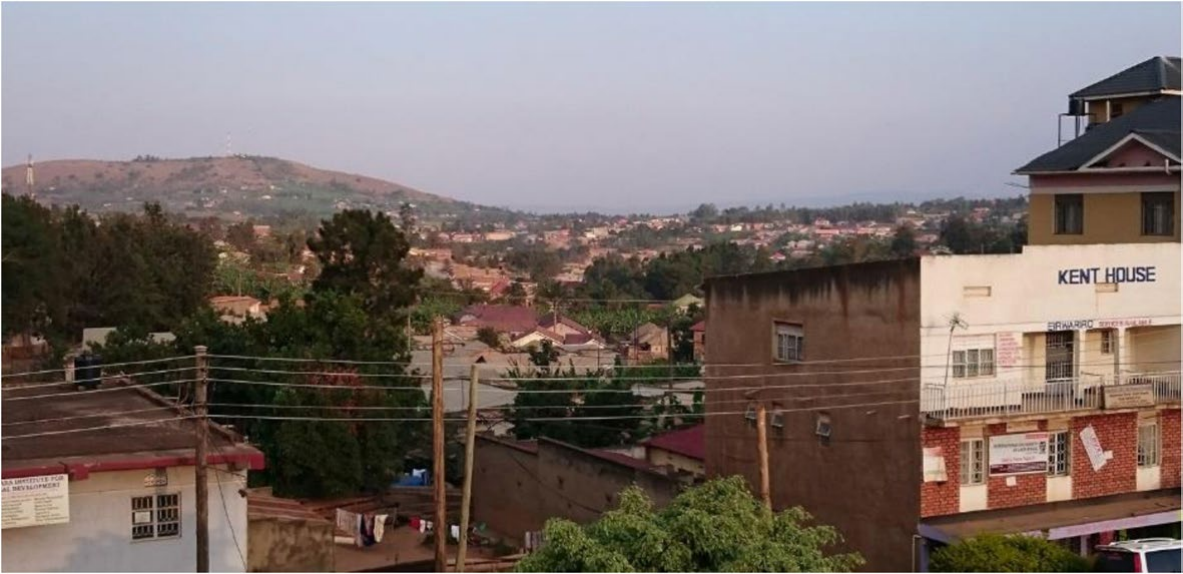
View over central Mbarara, 2018. Source: Mackay, H

Mbarara (Fig. 3) is the larger of the two sites and the one that has seen more investment. It is also a regional administrative hub. Its economic character comprises farming, marketing activities, and informal small-scale business (Habitat 2012). (ibid). The region is a known cattle-farming area with milk processing facilities (Mbarara Mbarara (2016). Arable farming concentrates on matooke, potatoes and millet (Habitat 2012) with some coffee production (MDLG 2015).

**Fig. 4:**
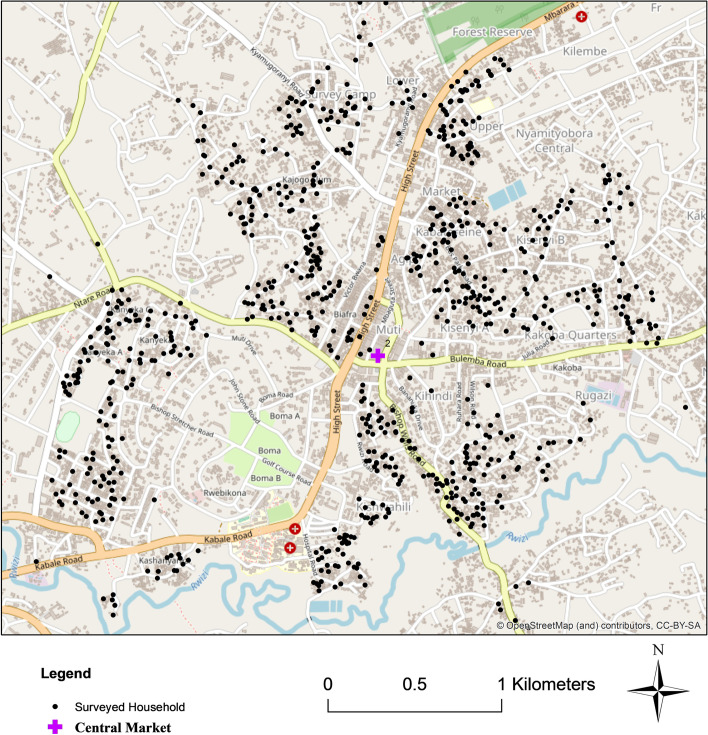
Map of Mbarara, Uganda. Dots show the households surveyed. Source: Open street maps. Created by Mackay, H., April 2022

The British established a civil station at Mbarara in 1906, officially proclaiming it a town (Weekes 1973), due to its good defensive position and opportunity for trade. It has remained a centre for regional trade and an important transportation node. The town’s linear spatial pattern extending north-east and south-west along the Kabale-Kampala road can be seen in Fig. 4. Analysis of the town’s developmental pattern since 1984 suggests rapid growth along this main artery between 1984–1999 (Brian 2016). Brian notes a still prevalent preference for land-use zoning and segregation of activities (Brian 2016).

## Findings from Mbale and Mbarara

Table 1 highlights some of the key findings from the household survey in Mbale and Mbarara on food sourcing, transfers, diet diversity, food insecurity and practicing farming. At the aggregated average, both cities had a majority of households with low or medium dietary diversity (HDDS score 4 in Mbale, 5 in Mbara, Table 1). The food groups consumed consisted predominantly of cereals (largely maize), roots and tubers (largely matooke), legumes (largely beans), and sugar at these low and medium categories (details not shown). A not insignificant proportion of the 40% of households in Mbale that had a low diet diversity, and the 20% of Mbarara households in this category, had consumed from just one food group in the 24-h prior to the survey, this being maize (data not shown). Follow-up FGDs and interviews confirmed the centrality of maize (usually in the form of posho–a mash or porridge of maizemeal flour with water). In times of stress this can be the only food in a day providing carbohydrate-rich calorific content but low nutritional value. When possible, posho was eaten with a bean-tomato stew. Matooke with beans, or rice with beans were also common meals.

**Table 1:** Household Food Environment and Involvement in Farming

	**Mbale** (*N* = 1020)	**Mbarara** (*N* = 967)
**Food Sourcing** [multiple responses possible therefore > 100%]	**N (%)**	**N (%)**
Source: Traditional food markets (city centre and neighbourhood)	868 (85)	866 (89)
Source: Supermarket	237 (23)	233 (24)
Source: Grow It	389 (38)	342 (35)
Source: Small shops, restaurants, take-aways	652 (63)	739 (76)
**Food Transfers** (within previous year)	**Households N (%)**	
Households receiving (majority from rural-based relatives)	631 (62)	473 (49)
**Eating In or Out** (main meal previous day)	**Household Members** N (%) (Mbale *N* = 4242; Mbarara *N* = 3231)	
Own home*	3712 (87)	2881 (89)
School or workplace	366 (9)	231 (7)
Restaurant, shop or take-away	90 (2)	73 (2)
**Snacks and Sugar Consumption** (previous week)	**Households N (%)**	
Fried snacks (someone consumed daily or more) Total	175 (17)	114 (12)
Sugar (someone consumed daily or more)	758 (74)	640 (66)
Sugar-sweetened beverages (SSBs) [someone consumed daily or more)	91 (9)	87 (9)
**Food Security**	**%**	**%**
HFIAP Food Insecure (%)	67	56
HFIAP Food Secure (%)	33	44
Mean HFIASS (0 = no lack, 27 = often a lack of food in previous month)	7.1 score	5.0 score
**Dietary Diversity**		
Mean HH Dietary diversity score (HDDS) (0 = no food 12 = maximum)	4.4 score	5.2 score
FAO Dietary Diversity Category (Number food groups eaten within 24-h)	**%**	**%**
Low dietary diversity (< 3 food groups)	40	20
Medium dietary diversity (4–5 food groups)	31	37
High dietary diversity (> 6 food groups)	29	43
**Involvement in some form of Farming**	**N (%)**	**N (%)**
Involved in agriculture (any location)	663 (65)	493 (51)
Not involved in any agriculture	343 (34)	465 (48)

^*^The majority who did not eat their main meal at home were children who ate at school

Source: compiled from (Mackay et al. 2017; Mackay 2019a, 2019b, 2022)

More than 85% of households in both cities sourced their food from traditional marketplaces and less that 24% used supermarkets (Table 1), and then only sporadically and for specific products (Mackay et al. 2017). We also see around half (Mbarara) to 63% (Mbale) households receiving food transfers–these were overwhelmingly from rural-based relatives (Table 1). Most family members ate their main meal at home (Table 1). Follow-up interviews and focus groups claimed few could afford going to restaurants, though there was evidence of students and the least well-off snacking on cheap energy-dense street food (Mackay 2019a, 2019b). Yet the survey data does not support common nutrition transition claims of snacking, fast-food consumption or sugar-sweetened beverages with fewer than 9% claiming someone consumed a soda in the preceding 24-h (Table 1). Sugar was commonly added to tea and coffee.

Regarding feeling food insecure, mean HFIASS was 7 in Mbale and 5 in Mbarara (maximum 27 would be extreme famine), yet the Household Food Insecure Access Prevalence (HFIAP),^2^ grouped into food secure/food insecure, suggests 56–67% of Mbarara-Mbale households felt food insecure (Table 1). Finally, more than half of households (65% in smaller Mbale) had been involved in agriculture in either a rural or urban area during the year. This farming relationship, while found even in lower classes, was particularly a strategy of the more food secure, the salaried, the longer-term city residents, and the urban elite, drawing from the in-depth interviews (Mackay 2019b).

Our anthropologic measurements revealed a concerning level of overweight and obesity where 26% of 1,248 measured adults in Mbale fell into the WHO category of overweight (BMI (25–29.99 kg/m^2^) and 14% were obese (> 30 kg/m2) (see (Mackay et al. 2017) for details). In Mbarara the figures were 28% and 22% respectively (948 adults) (ibid). These data were higher than national statistics at the time which recorded 22% overweight and just 9% obese (MoH 2014). We also found 12–22% of Mbale and Mbarara households having someone suffering from diagnosed diabetes, or hypertension and some form of heart disease or stroke (details not shown, see Mackay et al. (2017)). These data indicated a clear NCD presence, and we took this (together with city-level and national-level data on child undernutrition, stunting and wasting) to confirm a city-level double burden of malnutrition.

Our findings, triangulated across all our data types indicated that food sources (places where food was acquired) and dietary diversity were similar across interviewees, regardless of gender, age, class, race or interactions therein. We found that most variation could be explained by income/work status (some might call this class) differences (Mackay 2019a, 2019b). Less than half of surveyed households had a member earning a regular salary (ibid).

The main difference in food environments encompassed the varying food access strategies deployed by the food secure and the food insecure, and these were distinct and informative (Mackay 2019b). The more food secure residents had more stable employment (linked to income/class), were able to buy in bulk and stock food (they moved around to buy at cheaper prices, and they had storage space), and they had relational interaction with the rural (via farming and direct food transfers–rural natural and social capital) (ibid). The least food secure (those who were unemployed/had difficulty making a living) were not able to engage in such food access strategies due to lower and highly variable/insecure incomes, less mobility and limited access to land (Mackay 2019b). Bulk stocking of major products (maize flour, beans, rice, sugar) was described by all the better-off interviewees but few others (ibid).

The involvement in farming, even of the elite, is a finding revealing the continued prioritisation of land as security within the urbanising Ugandan context, as other researchers have highlighted (Reid 2017; Goodfellow 2017). The continued perception and valuation of land as a principal asset in Ugandan culture is apparent in how interviewees described their lives, their efforts and their ambitions (Mackay 2019a, 2019b). Mbale and Mbarara’s urban agriculture mainly took the form of gardening of a few leafy green vegetables, maize, matooke for own consumption, among the slightly better-off. There was little evidence of highly organised entrepreneurial market gardening for city markets. Of note was that the agricultural practice of urban inhabitants in the Mbale/Mbarara contexts, while slightly improving food security and reducing food outlays, did not translate into better dietary diversity than non-farm households (Mackay et al. 2023). We speculate this may be due to the focus on the same food crops (maize, beans, matooke, potatoes) commonly consumed. Other have found similar–see the systematic review of UA for food security and nutrition by Warren et al. (2015) and findings in Kampala from Yeudall (2007).

Our FGDs with healthcare professionals initially suggested classed and gendered ideas of bodies, diets and weight, with concepts such as the ‘provider man’ or the ‘lazy expectant housewife’ or the irresponsible man who doesn’t provide for the family, with an overall initial tendency to ‘blame’ women, as Warin (2015) also notes (Mackay 2019a), before a more nuanced debate evolved (Table 2).

**Table 2:** Evolving Discourses—Urban Housewife as a Proposed, but hotly debated, Reason for Rising Obesity

Margaret	But here, you are fine. You are at home. You have a maid. The man goes out, he bring everything. You are at home, you just eat and sleep [*participants are chuckling*]
Amaka	Eh! How many women do we have….of that kind?! [*more laughter*]

Source: (Mackay 2019a, Table 3, p14)

The initial conceptualisation was that the rich were fat, and housewives and mothers were fat, due to greediness, laziness or ignorance; and that it was only the poorest who had food insecure monotonous diets (Mackay 2019a). Indeed, this was the initial discourse, put forward particularly by a number of men in the focus groups. These young, educated, both single and married, stably employed men were themselves an embodiment of the dominant patriarchal, heterosexual, educated urban ‘norm’ of local discourse (refer to Hovorka (2012) and Wyrod (2008)). Indepth interviews provided rich and quite confronting data regarding livelihood strategies in the face of urban Ugandan insecurities of sugar daddies/mommies and transactional relationships where deliberate weight-gain through specific food consumption practices or weight-training or implant strategies (for both men and women) may be employed in order to secure a livelihood. Such arguments are provocative: ‘They speak of gendered and classed relationships, of who has power and who does not, and they raise ethical questions. How real or widespread such representations are, is not possible to tell from my data. What is relevant here is that these narratives of urban bodies and behaviours exist’ (Mackay 2022, 202).

These kinds of blame claims and stereotyping are influenced by behaviouralist approaches to health and give little credence to the social determinants of health (Marmot et al. 2008) nor to how little power and choice many but the most-resourced actually have over their diets, employment circumstances and physical activity (Hunter-Adams 2019). Yet, as the FGDs progressed and some of the women present began to raise their voices and relay their communities’ experiences, a more nuanced but less straightforward picture emerged. This was one whereby the somewhat better-off still consumed relatively monotonous diets and had food security concerns, and the less well-resourced were increasingly experiencing NCDs (Mackay 2019a). Our biographic interviews also revealed how salaried elites invested in (ofttimes rural) farming as a food security measure, and still shopped in traditional markets and local stores, and only bought a few products occasionally from Western-style supermarkets (Mackay 2019b). A feminist geographic analysis reveals and explores the role of local socio-cultural-political implications of concepts like food, work, rural, urban, femininity, masculinity (Nagar et al. 2002) and underlying assumptions people may hold. Our data showed how these can interact to influence understandings and interpretations of bodies, of food, of farming, of identities, and yet the very process of revealing, discussing can also at the same time liberate and co-create new and more nuanced understandings, as we found in our focus groups (Mackay 2019a).

Finally, the role of institutions in facilitating food security via providing access to land, or sometimes via food subsidies, was particularly apparent in the findings from Mbarara State Prison, but also seen in schools, churches, other institutions who allowed staff (or nearby communities) to farm their land, in both cities (Mackay 2019b). We regard ‘institutions’ as either state organisations such as prisons, hospitals, schools, administrative or research bodies, or non-state actors such as churches or other religious bodies, who often own swathes of land in and around African cities. As noted in Mackay (2019b), organs of local or national governance we class separately as ‘government’ even though they are also a form of ‘institution’. The institutions we connect with this term however do not have a key mandate for citizen welfare in the way government planning agencies do. We conclude that urban institutions have potential to help residents overcome limitations of gender, class, land assets, or household structure, to improve food security, nutritional status and well-being: to interfere, if you will, in food and nutritional transformations. A similar circumstance was found in secondary cities of Ghana (Mackay 2018). Herein lies potential for positive intervention.

In summary, our data suggests that food system transitions had not advanced far along predicted nutritional transition in these cities, but an epidemiologic transition was further ahead. Findings did not fit well conceptualisations that urban residents have more diverse and secure food environments, consume more processed foods/snacks, or abandon agriculture, in comparison to rural populations. The next sections assess findings against Kampala (as Uganda’s primary city) and other primary African cities.

## Analysis: comparing these secondary cities to primary city studies

This comparative section follows the themes of diet diversity, food insecurity, NCD experience and interpretations, food sources and access strategies, role of agriculture and of institutional land, with our conclusions being summarised in Table 3.

**Table 3:** Conclusions from Comparative Analysis with Kampala and other African Primary Cities

Mbale/Mbarara finding	Comparison summary
Diet diversity: Low-medium (but medium mainly added groups of less nutritional value such as sugar and oil). Strong maize and starchy staples focus	Similar
Food insecurity: Common feeling of being food insecure; Mean HFIASS Mbale: 7; Mbarara: 5; % HHs food insecure HFIAP Mbale: 67% Mbarara: 56%	Similar
NCD experience & surrounding discourse: prevalence of overweight, obesity, diabetes, hypertension similar to Kampala and other African cities, despite the high levels of food insecurity and low diet diversity. Discourses similar	Similar
Food Sources: Lack of advanced supermarketisation of resident food baskets	Similar in Kampala; May be different from other African cities
Food sources: Persistence of traditional markets, and small shops that give credit, and where a customer is also more able to negotiate on price, for majority of residents’ needs was apparent across diverse residents	Similar in Kampala; May be different from some other African cities
Food access strategies: The ways Mbale/Mbarara residents accessed food from different sources varied by class, assets, and links to rural land/relations	Some difference due to greater mobility & link to farming
Active in *rural* agriculture: Own agriculture (via own or hired labour or family labour in rural area) continues to be important for many Mbale and Mbarara residents, largely supplementing food basket but some sales	Different (need for more representative surveys, not those selecting only UA households)
Differing role of an *urban* agriculture: UA mainly yard gardening of maize/leafy vegetables for consuming, less market gardening; little evidence of zero-grazing/free-roaming livestock; nor of poultry/small meat-rearing	Different
Potential of institutions to improve food security, via facilitating access to land to farm for staff/ surrounding communities, or simply via subsidies/donations	Different in that smaller cities may have less land pressure, & institutions may retain greater land areas

### Diet diversity

Comparing Table 1’s data to Kampala, a number of studies have also measured low diversity diets in the capital such as for adolescent girls (Berg et al. 2018), or the latest national survey where only 11% of women and young children met minimum dietary requirements (GoU 2020). A review of 11 southern African secondary and primary cities by Frayne and colleagues found mean HDDS of 6 across all cities, but when split by food security experience, food secure households had a median HDDS of 8 while insecure households had median HDDS of 5 food groups (Frayne et al. 2014). In comparison to four other secondary cities in Ghana and Kenya, Ugandan cities do seem to have lower mean HDDS (Mackay et al. 2023). The importance of maize/rice/beans for food security, and a lower diet diversity for the region, is reported by others (Ngaruiya et al. 2017; Raschke and Cheema 2008; Pottier 2015). Some have also noted institutions relying on maize (Frayne et al. 2014), often growing it on their land (ibid). This was also found by Mackay (2018) in secondary Ghanaian cities. Having a maize/carb-rich staple that provides calorific satiation is similar across many African contexts (Frayne et al. 2014; Mackay et al. 2023).

### Food insecurity

Frayne et al.’s review of 11 southern African cities found similar prevalence of approx. 60% food insecure HFIAP (Frayne et al. 2014), though poorer communities were selected. Many have raised concern about an urbanisation of food insecurity in Africa, particularly in relation to seasons, price fluctuations and time for school-fees payments [see for example (Tacoli 2019; Ruel et al. 1998; Crush et al. 2012; Prain et al. 2010; Pottier 2015)]. Gerster-Bentaya, drawing from studies of Accra, Cape Town, Dar es Salaam, Dakar, Yaoundé, Nairobi and more, notes the commonness of food insecurity and some involvement in urban agriculture as a way of supplementing food (Gerster-Bentaya 2015). Our findings do suggest, unsurprisingly, a link between food insecurity and employment insecurity, as do others (Battersby et al. 2019; Crush et al. 2012). Having secure employment with a reasonably reliable, liveable salary was not the norm in Mbale/Mbarara. These findings on the commonness of informality and underemployment across urban Africa have been found by others (Dodman et al. 2017; Cobbinah et al. 2015). A number of works have highlighted the role of urban agriculture as a strategy to overcome food insecurity but who farms, where and in what way differs greatly by context (Masvaure 2015; Gallaher et al. 2013; Orsini et al. 2013; Lee-Smith 2010).

### NCD experience

The Ugandan national STEPS survey found average 9% obese among all urban adults (MoH 2014) which is lower than our findings in Mbale/Mbarara (Table 1). STEPS noted national urban prevalence of diabetes and hypertension as 4.8% and 25% respectively (note: not directly comparable to our household data). We find the NCD experience fairly similar to Kampala and to other African cities, despite possible differences in food sources and access strategies. We should also note the number of studies beginning to point out similar relatively high prevalence of hypertension (Sani et al. 2022), diabetes and obesity (Mayega et al. 2012; Steyn and Mchiza 2014) even in rural areas to caution against viewing NCDs as a strongly urban phenomenon. Rural residents have different food sources and access strategies than urban residents. This serves to caution against too-rigid a focus on built-environment and urban lifestyle causality. The link to underlying and historic/childhood (foetal origins theory) experience of poverty and food insecurity is increasingly recognised as a shared cause regardless of urbanity of current residence (Barker 1997; Warin 2015; Steyn and Mchiza 2014). These biological feedback mechanisms, as well as socio-cultural constructions around fatness or health (Mackay 2022) do not vary significantly by city size across regions.

As mentioned earlier, the discourse around NCDs was highly classed and gendered in Mbale/Mbarara. Others have reported similar debates and framings from, for example, Malawi (Riley and Dodson 2014, 2016), Botswana (Hovorka 2012), South Africa (Hunter-Adams 2019) and more generally (Warin 2015; Wyrod 2008). We conclude that such NCD discourse is unlikely to differ by city scale. Gendered, classed and otherwise differentiated reasonings and power geometries tend to be broadly more regionally formulated rather than so locally defined. It is important to engage with and to combat misleading discourses. Importantly, our findings showed how social constructions are malleable, and how they can be contested via debate and a plurality of voices, in order to come to more nuanced and grounded understandings. To us, this offers emancipatory opportunity and signals intervention points.

### Food sources

The similarity of diet composition and food sources across a range of statuses runs somewhat contrary to nutrition transition conceptualisations, often based themselves on studies of primary cities, and lends weight to other researchers’ cautions about local variation (Nichols 2017; Himmelgreen et al. 2014). It is difficult to find studies of primary cities with comparable data, however, we feel there are indications that larger cities offer less diversity of food sources than secondary cities. Certainly, primary cities have greater number of restaurants, fast foods, street foods, shops, supermarkets and traditional markets due to their size and geographic spread. However, number (greater numbers of venues within a category) and density of these is not the same as variation in *types* of food sources. What we mean by this is that the ability for primary city residents to source food via their own production (farming) or via transfers from rural villages and rural relatives may be less than for secondary city residents due to greater physical and social distance and higher transport costs, but further work is needed.

### Food sourcing from traditional markets and small shops rather than supermarkets

Others have also suggested that the role of supermarkets even in Kampala is not as advanced as in other African cities (Wanyama et al. 2019; Haggblade et al. 2016), especially when compared to the Southern African or even Kenyan context (Reardon et al. 2003). The western-style supermarket as the source of the majority of the family’s food through a one-week large shop does not seem to have made a strong in-road into Uganda at this stage. How this will evolve remains to be seen but for the moment it seems that our secondary cities match Kampala in this aspect but how similar or different Uganda is to other SSA countries is somewhat unclear.

### Food access strategies

The continuing importance of linkage to the rural for these secondary city residents emphasises the role of multi-spatiality for more food secure urban lives. This link to rural land and to rural relations occurs for larger cities but may be weaker, or less common, or less easily maintained, but more research is needed (Foeken and Owuor 2001). Multi-spatial livelihoods may be less possible within large cities due to land access challenges, and due to greater distances, costs and transportation challenges to rural areas or to other food markets, suggesting that this may be an even more elite strategy (Jayne et al. 2003) in primary cities than in secondary cities where access to the rural is faster, easier, cheaper (Mackay 2018). That the better-off were able to buy in bulk and make cost savings and draw from other assets whilst food insecure individuals spent more on small amounts, has been found by others (Battersby and Watson 2019; Dodman et al. 2017).

### Active in rural agriculture

Our finding regarding the role of land and the continued importance of rural agriculture and of rural relationships contrast somewhat with earlier work from African primary cities such as Accra, Addis Ababa, Cape Town, Dar es Salaam, Kampala and Nairobi where the emphasis was around the urban poor’s farming of the city environment, see for example chapters in Egziabher et al. (1994) or Sanyal’s investigation of Lusaka residents (Sanyal 1985) and of course Maxwell, Levin and Csete’s work in Kampala (Maxwell et al. 1998). Farming in a rural area may be more prevalent and more significant for secondary than for primary city residents due to a shorter physical distance to rural land and rural relatives, lower transportation costs, and perhaps due to closer generational contact and relationship maintenance. Others do find evidence, however, of urban elites in capital cities in Kenya and Rwanda for example investing their salaries into buying rural land assets but this may be less accessible for urban poor (Jayne et al. 2003; Goodfellow 2017).

### Differing role of an *urban* agriculture

There are many different types of UA (backyard gardening, or open space/public land allotment style farming or grazing, or clearly market-oriented vegetable gardening) taking place in different tenure arrangements and contexts, see Mackay’s (2018) discussion of this. With organised market gardening, studies of UA in primary African cities abound, such as Accra (Agbeko 2010; Asomani-Boateng 2002; Maxwell 2000), Dar es Salaam (Schmidt et al. 2015; Drechsel and Dongus 2009), Nairobi (Prain et al. 2010). Some found that UA in capitals was generally practised by recent rural migrants or the poorest or most vulnerable residents (Mougeot 2006; Asomani-Boateng 2002). Others found it more a practice of longer-term and middle-class residents as they were the ones who had the ability to access land, or found indications that it was becoming more elitist (Lee-Smith 2010; Mbiba 2001).

There has been a tendency for literature investigating intensive market gardening UA by community groups (sometimes migrant groups) on public-owned land for the large and proximate urban consumer base in many primary cities (such as Accra, Kumasi, Kampala, Dar es Salaam; Nairobi etc.) to make generalising claims for all urban agriculture when really this is a specific activity in specific places for specific peoples (Prain et al. 2010; Lee-Smith 2010; Drechsel and Dongus 2009; MOFA 2007; Mbiba 2001). We did not find such kind of large market gardening sites in Mbale and Mbarara. We speculate that smaller cities do not have the market to sustain such and there is thus a city-scale difference here.

The backyard gardening of a few leafy vegetables or starchy staples (as we saw in Mbale/Mbarara) as a food basket supplementation has been found by other studies in primary cities (Prain et al. 2010; Egziabher 1994; Armar-Klemesu et al 2000; Maxwell 2000). It is difficult, however, to assess how common and widespread such gardening was since many of these studied specifically sampled farm households. Yet in the 11 Southern African cities studied by AFSUN, UA was not a significant practice (Crush et al. 2012). We speculate that as city size, density and thus land-use pressure increases in larger cities, having land next to the home in which to garden may be affordable to fewer people. These findings on the commonness, type and practitioner of UA are important secondary city differences and represent an interesting contribution to the literature.

### Rural–urban links and multi-spatiality

The importance of assets, particularly rural-based assets and rural social networks in supporting food security for Mbale/Mbarara residents is a finding that does not seem to be replicated as clearly in studies from primary African cities. Many of previously cited studies of primary city residents’ relation to agriculture focused on urban land and urban farming and thus paid less attention to urban residents farming in rural areas. These findings support work highlighting the strong connection between urban and rural areas and multi-spatiality as a food access strategy (Berdegué et al. 2014; Satterthwaite et al. 2010; Bah et al. 2003; Foeken and Owuor 2001). We hypothesise that there may be city-scale differences influencing urban residents of primary cities’ engagement in both a rural and an urban agriculture. For example, a greater proportion of primary city residents may have less contact with rural land and rural people than secondary city residents, or due to the often-greater physical distance in reaching rural areas from large cities than from many of SSA’s smaller cities. Yet perhaps primary cities may house a greater proportion of new salaried elite who invest in rural land as Jayne notes (2003). Such city scale difference is intriguing, requires further investigation, and may contain interesting insights as we consider who will farm the future more urbanised, secondary African city landscapes.

### The potential of institutions

There may be a city-scale difference in institutions’ abilities to support food security, but further research is necessary. Yap noted this role of institutional land in Kampala though this was not always legal nor accepted in city ordinances (Yap 2013). A number of larger cities in Ghana (Kumasi, Accra, Tamale), Tanzania and Kenya also document UA on institutional land, though principally as intensive market gardening for sale (Prain et al. 2010; Lee-Smith 2010; Drechsel and Dongus 2009; MOFA 2007; Mbiba 2001). Institutions supporting food security and/or land access is mapped in other secondary cities of Ghana (Mackay 2018) and in the capital Accra (Flynn-Dapaah 2002). We speculate that institutional land may be under less intensive development pressure in secondary cities than in primary cities, which may allow institutions greater power to respond to local needs. This is a significant contribution to literature on both urban agriculture and urban food security, and to debates on local power dynamics and land tenure as Mbiba also notes is his overview of primary cities in Eastern and Southern Africa (Mbiba 2001).

These main similarities and differences, identified from this analysis are summarised in Table 3 above.

## Reflections

This section reflects on our comparative analysis and some of the possible implications of our findings.

### Slower, more hybridised food-system change in secondary cities

The most food insecure Mbale/Mbarara residents were not simply income-poor: they were also land-poor, social relations-poor, and asset-poor (rural and urban assets). The findings around food sourcing and food access strategies across diverse personal circumstances contrasts with some nutrition transition claims and urban development theories which suggest that urban life facilitates greater human, social, financial and physical assets (Turok and McGranahan 2013; Popkin et al. 2012; Popkin 2001; Ruel et al. 1999). In this way these secondary Ugandan cities suggest, similarly to studies from primary African cities, that urban residence alone is no indicator of secure food access (Satterthwaite et al. 2010). The not very advanced inroad of the Western-type supermarket in Uganda, highlights the value in cities supporting traditional open- or covered-marketplaces for access to diverse and healthy foods (Wanyama et al. 2019). The consideration of which urban residents engaged in agriculture, and where and in what form, suggested some possible difference from primary African city studies.

### The urban is dependent on the rural, perhaps even more so in secondary cities

Having active rural relations was associated, in our data (both qualitatively and statistically), with a better standard of, and certainly a more food secure, urban Mbale and Mbarara life. Relations matter; geography matters. As Pieterse and Parnell note, ‘African cities have several distinctive features. First, they are integrally connected to rural areas through the practice of circular migration, a strategy for maintaining multiple bases so as to optimise livelihoods and mitigate the risks of settling permanently in economically, environmentally, socially or politically precarious African towns’ (Parnell and Pieterse 2014, 9). Our findings indicate that these rural–urban relations and the ability to practice multi-spatial livelihoods, were influenced by socio-economic status, being more common and productive among those who were more food secure and had salaried employment. We see indications that such intersections may be somewhat less in larger cities, but more research is necessary.

This urban–rural interaction may also complicate the nutrition transition model (Popkin 2001; Popkin et al. 2012), which has tended to view the urban in isolation and as a cause in itself of food and epidemiologic transitions. This urban–rural interdependence supports a more recent body of work that challenges the idea of separation, of binaries and of separate states to transit between. As Riley and Dodson note: ‘Discourse about the “nutrition transition” taking place in African cities continue to be implicitly rooted in binary categories of foods characterised as urban and rural, modern and tradition, global and local’ (Riley and Dodson 2016, 53). We would broaden their claim from simply ‘food’ to incorporate food sources and access strategies, including farming. Lifestyle change whereby urban elites may be becoming city-based sedentary farmers also complicate binary associations of city = non-agricultural and rural = agricultural.

Our explorations of individuals’ daily spatial practices (Massey 2005; Lefebvre 1974) regarding food and farming highlight the interactions within and between urban space(s) and other urban or rural places. This supports the theorising of the socially constructed, and relative and relational nature of space (Massey 2005; Lefebvre 1974). The specific socio-spatial imaginaries focus group participants and individual interviewees offered of, for example, the role of agriculture, or of obese bodies and their behaviours, revealed some Ugandan discourses around people, places and activities, as well as how embedded local power relations are. Such excavation of social constructions and underlying, often unquestioned, gendered and classed power dynamics is very much in the spirit of feminist geographic analyses. We see these discourses and interpretations as similar across city scales. Our findings also emphasise how discourses constantly evolve, and thus can be contested or re-shaped. To us, this represents the possibility of a pathway towards progressive and emancipatory strategies (Parker 2016) that could be employed to reduce food, farming and health inequalities.

### Need to re-evaluate food system-nutritional-NCD relations

Our study raises questions of causal mechanisms around the evident non-communicable disease burden and prompts consideration of what other factors may be driving the epidemiologic transition in Uganda, which is also not confined to only urban populations. These other drivers likely include physical activity versus energy intake imbalances, but may also include diet quality and nutritional content, level of processing, pollutant loads, as well as cooking practices, oil used, or portion sizes, feast-famine eating patterns, or genetic susceptibilities (Bray 2004). Future research needs to consider these wider aspects of food and nutritional environment and any transformation therein. Other drivers of nutritional and epidemiological change are influenced by national policies, pricing, advertising, trade relations and thus also less likely to differ by location within a country. Local urban environments, however, could differ in how they shape their physical infrastructure, ability to walk or cycle or exercise in public space, their transportation possibilities, and here there is scope for city-scale and place-specific differences. An important implication of our findings could be to reframe the NCD discourse. Suffering NCDs is not due to poor decision-making or bad behaviour, as some may initially assume. NCDs, particularly obesity, are influenced by a lack of power and resources to be able to make healthy food and lifestyle choices, rather than by ignorance or lack of will (Warin 2015; Marmot et al. 2008). Such social determinants of health, and socio-cultural interpretations of food, of bodies, and behaviours, are unlikely to display variation by city-scale, though local context and dynamics may shape local expressions to some degree.

## Closing comments

Our analysis of how our Mbale/Mbarara findings relate to previous studies of Kampala and of other large African cities suggest a good deal of similarity of food insecurity, dietary diversity, and of NCD experiences and understandings. The main difference was around the food access strategies, access to land within the city for individuals and institutions, and the engagement with agriculture and interaction with the rural. How this might change as these secondary cities grow further is not clear but there should not be an assumption that primary city experiences will inevitably be followed. The findings highlight the need to contextualise nutrition transition theory to place, forcing us to recast the urban spaces of Mbale and Mbarara on relative and relational links to the rural. The most food secure urban lives in these Ugandan secondary city socio-economic and political contexts relied upon the rural, as well as salaried employment. In comparison to studies from Kampala and other primary cities within SSA, or studies from national-level data sets, our findings suggest less advance along theorised food, farming and nutritional transitions in these secondary cities, yet comparable experience of NCDs. This is intriguing, has possible implications for theory, and certainly warrants further research.

In terms of implications for policy, for practice, for future research, our findings support those recently calling for viewing cities through a food lens (Battersby and Watson 2019). Traditionally, urban planners and governors have not considered food security nor diet quality as within their remit focusing instead on the usual ‘urban priorities such as public transportation and decent housing’ as stated by the Assistant Director-General of FAO’s Economic and Social Development Department in the 2018 UCL/FAO report on integrating food into urban planning (Cabannes and Marocchino 2018, v). Yet the commonness of feeling food insecure, found in numerous African cities (Battersby and Watson 2019; Frayne et al. 2010) regardless of their location within the urban hierarchy (including Mbale and Mbarara) across a diversity of gendered, classed and otherwise differentiated circumstances, should necessitate action from planners and policymakers. Dietary insecurity can be seen as both an outcome, and a driver, of income insecurity (Battersby and Watson 2019; Crush et al. 2012). An obvious though not necessarily easy to implement policy implication would be to work toward improving the employment situation in African cities, regardless of their size/status. As other researchers have noted, though not all are in agreement, there is evidence of urban population growth in the absence of employment growth in many parts of SSA: ‘jobless growth (along with increasing poverty) is taking place in many parts of the continent’ (Battersby and Watson 2019, 18). Our findings emphasis how the unreliability of incomes and the widely experienced under-employment, and informality of employment, are serious obstacles to urban residents’ power over their livelihood strategies and their ability to affect their food security and dietary circumstances. Urban authorities ought to prioritise employment creation clearly. However, beyond this, they could modify their sometimes-punitive approach to informal livelihoods, and they could move away from policies and actions that discourage of (urban)farming as a livelihood security, or that infringe upon active rural–urban links. In addition, local authorities and relevant stakeholders could work to remove obstacles to urban food flows and to the diverse resident food sourcing strategies. A proactive engagement with local institutions on how urban land can be used to benefit adjacent communities is an area of underexplored opportunity. These strategies are relevant, regardless of the city-scale, as indeed others have also highlighted (Kinyanjui 2014; Crush and Frayne 2011; Lindell 2010; Skinner 2019), however our findings suggest that there may be greater potential for positive impact of such approaches at the secondary city scale.

## Data Availability

The data upon which our findings are based are available in open access research publications. Access to interview and focus group transcripts, and household survey data are not publicly available due to privacy protection requirements, however some elements of these may be available upon request.

## Abbreviations

AFSUN: African Food Security Urban Network
BMI: Body Mass Index
FAO: Food and Agriculture Organization
FGD(s): Focus Group Discussion(s)
GIS: Geographic Information System
HDDS: Household Dietary Diversity Score
HFIAP: Household Food Insecurity Access Prevalence
HFIASS: Household Food Insecurity Access Scaled Score
NCD(s): Non-communicable disease(s)
SSA: Sub-Saharan Africa
UA: Urban Agriculture
UN: United Nations
WHO: World Health Organization

## Footnotes

1 the World Population Review online database cites 76,400 for Mbale and 97,500 for Mbarara in 2019 (WPR 2019).

2 See Coates and Bilinsky (2007) for further more on HFIASS/HFIAP and Swindale and Bilinsky (2006) for HDDS.
